# Rhabdomyosarcoma of the biliary tract in a child: a case report

**DOI:** 10.3389/fped.2024.1436446

**Published:** 2024-08-07

**Authors:** Tang Ran, Chen Gong, Dong Rui, Zheng Shan

**Affiliations:** ^1^ Department of Pediatric Surgery, Anhui Provincial Children's Hospital, Hefei, China; ^2^ Shanghai Key Laboratory of Birth Defect, Department of Pediatric Surgery, Children's Hospital of Fudan University, Shanghai, China

**Keywords:** rhabdomyosarcoma of the biliary tract, pediatric, ERCP, surgery, chemotherapy

## Abstract

Pediatric rhabdomyosarcoma of the biliary tract (BRMS) is extremely rare. Here, we present a case of a 2-year-old child who was initially misdiagnosed with choledocholithiasis upon admission. The diagnosis was later confirmed as BRMS through endoscopic retrograde cholangiopancreatography (ERCP). The patient was cured through surgery followed by chemotherapy. Due to the lack of specific early symptoms and the challenges in imaging differentiation, particularly in pediatric patients, clinical awareness of this condition needs to be heightened. Our findings indicate that ERCP is currently the optimal diagnostic tool for this disease, and a combination of surgery and chemotherapy can yield better therapeutic outcomes.

## Introduction

Rhabdomyosarcoma (RMS) is the most common malignant tumor in children, the incidence rate in the American population is 0.4 cases per 100,000 people, biliary tract rhabdomyosarcoma (BRMS) accounts for only 0.5% of all RMS (1). BRMS is the most common malignant cause of obstructive jaundice in pediatric patients. The tumor can originate from any part of the intrahepatic and extrahepatic biliary tract, with the common bile duct being the most frequent primary tumor site (2). Due to the low incidence of BRMS, there is limited literature available, primarily derived from case reports. Diagnosing BRMS is highly challenging, and there is a lack of convincing treatment guidelines. In particular, the role of surgery in the treatment of patients with BRMS has become controversial in recent years, with some studies questioning the necessity of aggressive surgical resection for these tumors (1). Nevertheless, chemotherapy is considered to have a significant effect on BRMS both before and after surgery (3). Our case report aims to enhance clinicians’ awareness of this type of tumor. For BRMS that is difficult to differentiate through imaging, ERCP is the best diagnostic method. For resectable early-stage BRMS, surgery combined with chemotherapy is a reasonable treatment approach.

## Case report

**Figure 1:**
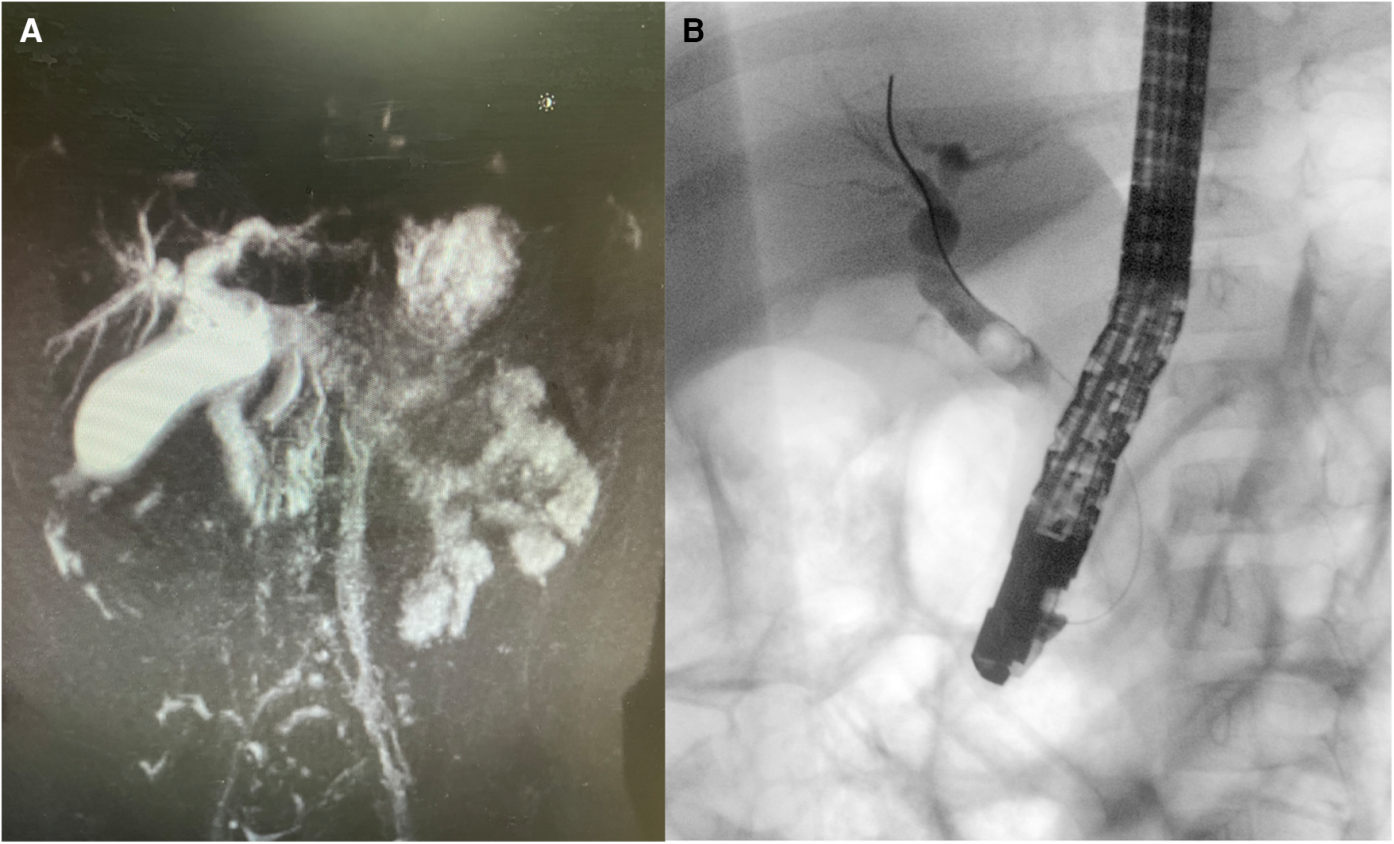
(**A**) Abdominal CT suggests dilation of the common bile duct and hepatic duct; (**B**) ERCP revealed a mass in the mid-common bile duct, and a biopsy confirmed it as tumor tissue.

A two-year-old female patient presented to our hospital with a one-month history of recurrent intermittent abdominal pain. Physical examination revealed that she had no jaundice or scleral icterus and no signs of anemia. Abdomen was soft without any palpable masses, there was tenderness in the right upper quadrant, and neither the liver nor the spleen was palpable. Abdominal CT suggests dilation of the common bile duct and hepatic duct (Figure 1A). Laboratory tests showed no abnormalities in infection markers and liver function, tumor marker tests indicated: AFP 1.04 ng/ml, CEA 3.18 ng/ml, Ferritin 50.75 ng/ml, NSE 21.57 ng/ml.

**Figure 2:**
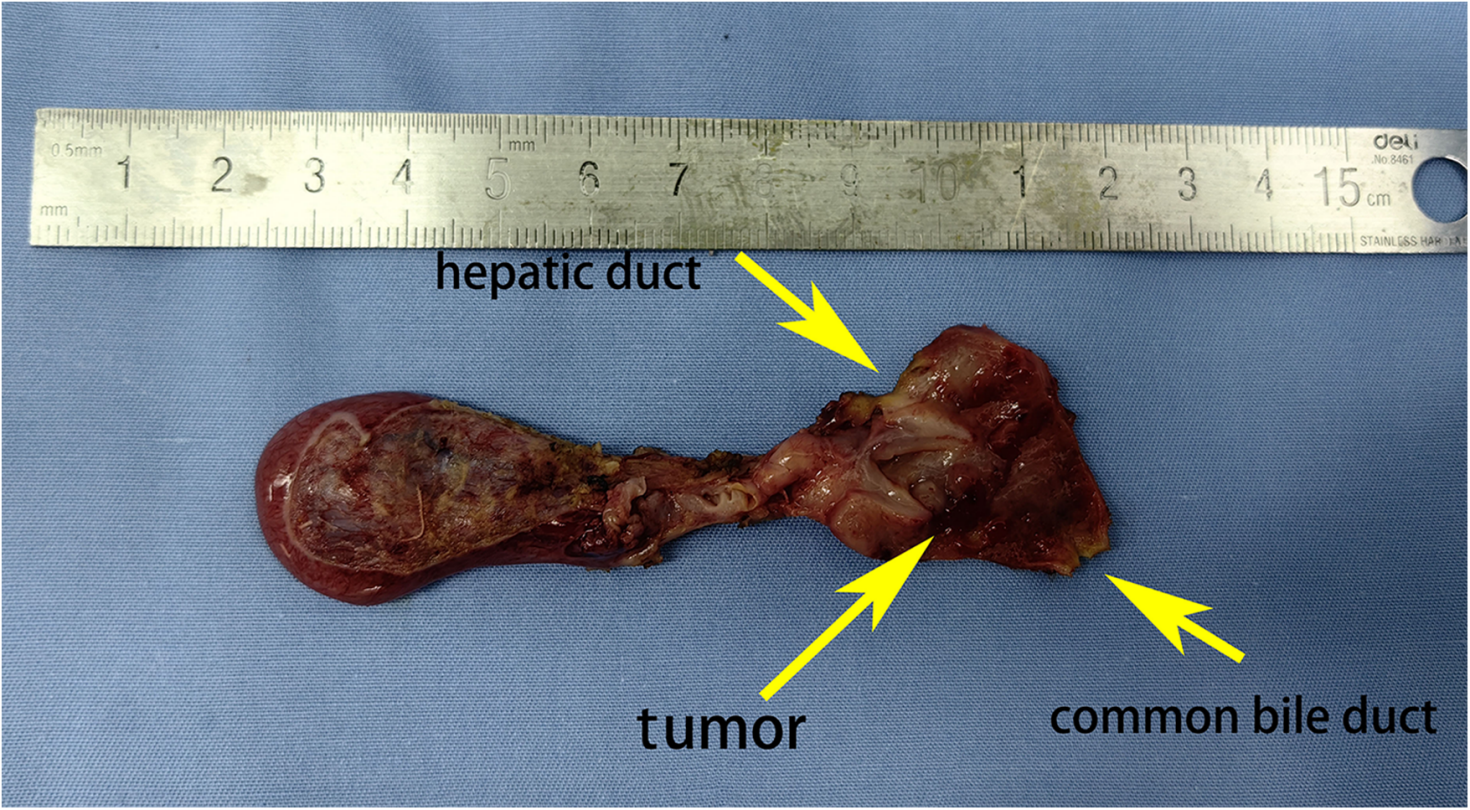
Surgical specimen: tumor located in the middle segment of the common bile duct, presenting a grape-like appearance.

**Figure 3:**
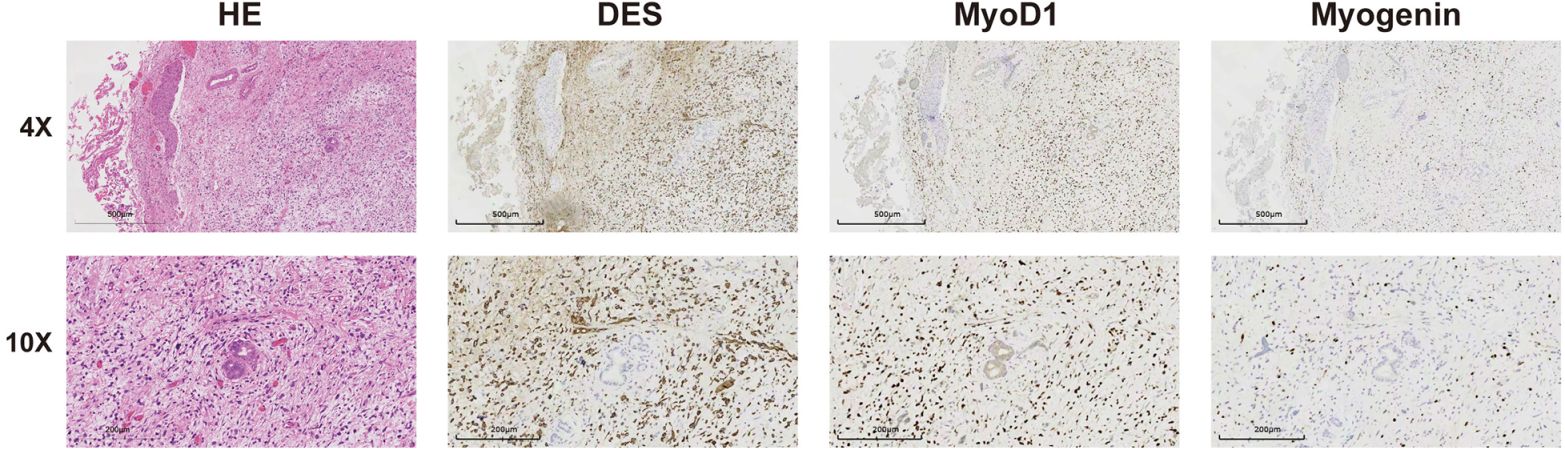
The microscopic appearance and immunohistochemistry of biliary rhabdomyosarcoma results suggest DES(+), MyoD1(+), myogenin(+). The final pathology confirmed “embryonal rhabdomyosarcoma”.

The patient underwent an ERCP examination, during which we discovered an occupying lesion in the common bile duct (Figure 1B). Contrary to expectations of a stone, we found a tumorous mass. A portion of the tumor tissue was sampled during the endoscopic procedure and sent for frozen pathology, which reported “Spindle cell malignant tumor.” Subsequently, a PET- CT scan was performed, revealing no lymph node metastasis or distant organ metastasis. The patient then underwent laparoscopic excision of the common bile duct lesion, hepaticojejunostomy (Roux-en-Y), and lymph node dissection. The excised surgical specimen showed a tumor in the middle segment of the common bile duct (Figure 2). Pathological results confirm “embryonal rhabdomyosarcoma” (Figure 3). According to the staging system established by the Intergroup Rhabdomyosarcoma Study Group (IRS) (4), it was classified as TNM Stage I, IRS Group I. The patient underwent chemotherapy with the VAC regimen, which included vincristine, actinomycin D, and cyclophosphamide, during the six-month follow-up after surgery, no recurrence or metastasis of the tumor was observed.

## Discussion

Obstructive jaundice is the most common clinical manifestation of BRMS (5). However, This child was admitted due to abdominal pain. Based on imaging examinations, we initially misdiagnosed it as choledocholithiasis (Figure 1A). There have been reports of patients initially misdiagnosed with choledochal cysts who were later found to have BRMS during surgery (6). The possibility of biliary tumors should be considered in the differential diagnosis of unexplained abdominal pain and obstructive jaundice. ERCP and biopsy are currently considered the most accurate and effective diagnostic methods (7). ERCP, in particular, is the preferred examination for BRMS as it allows for a definitive diagnosis and tissue biopsy, with a lower risk compared to percutaneous biopsy (8). However, there have also been reports of uncontrollable bleeding caused by ERCP, requiring emergency surgery (9).

Surgery aims to completely remove the tumor while maintaining negative margins (R0 resection) (1). For patients who cannot achieve R0 resection, neoadjuvant chemotherapy or local radiotherapy is considered to provide better efficacy. Some reports have indicated that complete remission and long-term survival of BRMS can be achieved without any surgical tumor resection (10, 11). However, more studies suggest that the absence of surgical resection is significantly associated with higher recurrence rates and has been proven to be an independent risk factor for mortality. Delayed surgery following chemotherapy for tumors that cannot be completely resected in the first instance has been shown to provide significant benefits (2, 12). The resection of extrahepatic bile ducts with biliary enteric, is the most commonly used surgical method and has been proven to be a safe procedure with good long-term outcomes in children (13). For low-risk group Stage I patients, postoperative VAC regimen excluding irinotecan is recommended (14). Patients who undergo incomplete Stage I or II resection require radiotherapy, whereas it is not used for those who initially achieve complete resection (R0 resection) (1).

Fuchs et al. (15) indicate that the 5-year overall survival (OS) rate for patients treated after the year 2000 is 65%. Current results show that the mortality rate for pediatric patients with BRMS remains quite high. Due to the rarity of the tumor, there is a lack of sufficient case numbers and further summary analysis for standardized treatment. We hope that our report of successful treatment for this patient can provide a valuable case basis for subsequent research.

## Conclusion

Pediatric BRMS is extremely rare, however, it should be included in the differential diagnosis of unexplained abdominal pain and jaundice. Clinicians should enhance their awareness of this condition to avoid misdiagnosis and unnecessary surgical trauma. Our case report indicates that compared to imaging studies, ERCP serves as a valuable tool for diagnosing this disease, providing crucial information for confirmation. In treating this condition, it is crucial to determine the disease stage. A comprehensive treatment approach, combining surgery with chemotherapy, typically yields favorable treatment outcomes.

## Data Availability

The original contributions presented in the study are included in the article/Supplementary Material, further inquiries can be directed to the corresponding authors.

## References

[1] Urla C Warmann SW Sparber-Sauer M Schuck A Leuschner I Klingebiel T Treatment and outcome of the patients with rhabdomyosarcoma of the biliary tree: experience of the cooperative weichteilsarkom studiengruppe (CWS). BMC Cancer. (2019) 19(1):945. 10.1186/s12885-019-6172-5 31610788 PMC6791000

[2] Guerin F Rogers T Minard-Colin V Gaze MN Terwisscha S Van Noesel M Outcome of localized liver-bile duct rhabdomyosarcoma according to local therapy: a report from the European paediatric soft-tissue sarcoma study group (EpSSG)-RMS 2005 study. Pediatr Blood Cancer. (2019) 66(7):e27725. 10.1002/pbc.27725 30920113

[3] Castellino S Muir A Shah A Shope S McMullen K Ruble K Hepato-biliary late effects in survivors of childhood and adolescent cancer: a report from the children’s oncology group. Pediatr Blood Cancer. (2010) 54(5):663–9. 10.1002/pbc.22265 19890896 PMC2838980

[4] Rudzinski ER Anderson JR Hawkins DS Skapek SX Parham DM Teot LA. The world health organization classification of skeletal muscle tumors in pediatric rhabdomyosarcoma: a report from the children’s oncology group. Arch Pathol Lab Med. (2015) 139(10):1281–7. 10.5858/arpa.2014-0475-OA 25989287 PMC4651658

[5] Shen CH Dong KR Tao YF Chen G Li RD Zhang QB Liver transplantation for biliary rhabdomyosarcoma with liver metastasis: report of one case. Transplant Proc. (2017) 49(1):185–7. 10.1016/j.transproceed.2016.11.028 28104133

[6] Tripathy TP Patidar Y Chandel K Varadarajan A Sood V Laroia ST. Embryonal rhabdomyosarcoma of the biliary tree as a differential in a paediatric patient presenting with biliary dilatation: not always a choledochal cyst. Acta Med Litu. (2022) 29(1):112–7. 10.15388/Amed.2021.29.1.2 36061928 PMC9428640

[7] Kirli EA Parlak E Oguz B Talim B Akcoren Z Karnak I. Rhabdomyosarcoma of the common bile duct: an unusual cause of obstructive jaundice in a child. Turk J Pediatr. (2012) 54(6):654–7. 23692794

[8] Scottoni F De Angelis P Dall'Oglio L Francalanci P Monti L de Ville DGJ. ERCP with intracholedocal biopsy for the diagnosis of biliary tract rhabdomyosarcoma in children. Pediatr Surg Int. (2013) 29(6):659–62. 10.1007/s00383-013-3282-z 23417547

[9] Perera MT McKiernan PJ Brundler MA Hobin DA Mayer DA Mirza DF Embryonal rhabdomyosarcoma of the ampulla of vater in early childhood: report of a case and review of literature. J Pediatr Surg. (2009) 44(2):e9–11. 10.1016/j.jpedsurg.2008.10.113 19231522

[10] Pater L Turpin B Mascia A. Pencil beam scanning proton therapy for rhabdomyosarcoma of the biliary tract. Cureus. (2017) 9(10):e1747. 10.7759/cureus.1747 29226038 PMC5716683

[11] Spunt SL Lobe TE Pappo AS Parham DM Wharam MJ Arndt C Aggressive surgery is unwarranted for biliary tract rhabdomyosarcoma. J Pediatr Surg. (2000) 35(2):309–16. 10.1016/s0022-3468(00)90030-7 10693686

[12] Lautz TB Chi YY Li M Wolden SL Casey DL Routh JC Benefit of delayed primary excision in rhabdomyosarcoma: a report from the children’s oncology group. Cancer-Am Cancer Soc. (2021) 127(2):275–83. 10.1002/cncr.33275 PMC7790947 33079399

[13] Yeung F Fung A Chung P Wong K. Short-term and long-term outcomes after roux-en-Y hepaticojejunostomy versus hepaticoduodenostomy following laparoscopic excision of choledochal cyst in children. Surg Endosc. (2020) 34(5):2172–7. 10.1007/s00464-019-07004-5 31342261

[14] Aye JM Xue W Palmer JD Walterhouse DO Arnold MA Heaton TE Suboptimal outcome for patients with biliary rhabdomyosarcoma treated on low-risk clinical trials: a report from the children’s oncology group. Pediatr Blood Cancer. (2021) 68(4):e28914. 10.1002/pbc.28914 33501771 PMC8765674

[15] Fuchs J Murtha-Lemekhova A Kessler M Gunther P Fichtner A Pfeiffenberger J Biliary rhabdomyosarcoma in pediatric patients: a systematic review and meta-analysis of individual patient data. Front Oncol. (2021) 11:701400. 10.3389/fonc.2021.701400 34660271 PMC8515851

